# Sex Differences of Uncinate Fasciculus Structural Connectivity in Individuals with Conduct Disorder

**DOI:** 10.1155/2014/673165

**Published:** 2014-04-14

**Authors:** Jibiao Zhang, Junling Gao, Huqing Shi, Bingsheng Huang, Xiang Wang, Weijun Situ, Weixiong Cai, Jinyao Yi, Xiongzhao Zhu, Shuqiao Yao

**Affiliations:** ^1^Shanghai Key Laboratory of Forensic Medicine, Institute of Forensic Science, Ministry of Justice, No. 1347 West Guangfu Road, Shanghai 200063, China; ^2^Medical Psychological Institute, The Second Xiangya Hospital, Central South University, No. 139 Middle Renmin Road, Changsha, Hunan 410011, China; ^3^Department of Medicine, LKS Faculty of Medicine, The University of Hong Kong, No. 102 Pokfulam Road, Hong Kong; ^4^Centre of Buddhists Studies, The University of Hong Kong, No. 102 Pokfulam Road, Hong Kong; ^5^Department of Biomedical Engineering, School of Medicine, Shenzhen University, No. 3688, Nanhai Ave, Shenzhen, Guangdong 518060, China

## Abstract

Conduct disorder (CD) is one of the most common behavior disorders in adolescents, such as impulsivity, aggression, and running from school. Males are more likely to develop CD than females, and two previous diffusion tensor imaging (DTI) studies have demonstrated abnormal microstructural integrity in the uncinate fasciculus (UF) in boys with CD compared to a healthy control group. However, little is known about changes in the UF in females with CD. In this study, the UF was illustrated by tractography; then, the fractional anisotropy (FA), axial diffusivity, mean diffusion, radial diffusivity (RD), and the length and number of the UF fiber bundles were compared between male and female patients with CD and between female patients with CD and female healthy controls, as well as between males with CD and healthy males. We found that males with CD showed significantly higher FA of the bilateral UF and significantly lower RD of the left UF when comparing with females with CD. Meanwhile, significantly higher FA and lower RD of the bilateral UF were also found in boys with CD relative to the male healthy controls. Our results replicated previous reports that the microstructural integrity of the UF was abnormal in boys with CD. Additionally, our results demonstrated significant gender effects on the UF of patients with CD, which may indicate why boys have higher rates of conduct problems than girls.

## 1. Introduction

Conduct disorder (CD) is a behavioral disorder related to impulse control that is diagnosed in individuals before 18 years of age. Patients with CD display severe aggression, actions that inflict pain on or deny the rights of others, and a history of status offenses [1]. CD has been reported to occur in about 16% of preadolescents [2]. It incurs a large social cost, as CD is almost always a prognosticator of antisocial personality disorder (APD) in adulthood [3].

Several methods, such as structural and functional magnetic resonance imaging (sMRI and fMRI) and diffusion tensor imaging (DTI), have been used to measure neurobiological activity in CD. Specific cortex areas or the fiber bundles connecting them have been shown to be impaired or dysfunctional in CD patients comparing with healthy controls. For example, in sMRI studies, volumes of the prefrontal cortex (PFC) (including the orbitofrontal cortex [OFC]) [4], temporal cortex [4], anterior cingulated cortex [5], amygdala [4, 6, 7], and insula [6, 7] were decreased in CD males when compared to healthy controls. Further studies revealed that, comparing to male healthy controls, the cortex thickness of CD males was reduced in brain regions that include the superior temporal and parietal lobes [8].

Results from fMRI research demonstrated abnormal activation in the OFC [9–11], ventromedial PFC [12], anterior cingulated cortex [13], and amygdala in CD relative to healthy controls. Interestingly, the amygdala activity was reduced in CD with callous-unemotional trait [14, 15] but increased in CD without callous-unemotional trait [9, 15, 16]. Meanwhile, studies on intermittent explosive disorder and destructive behavior disorder showed a dysfunctional connectivity between the OFC and amygdala [9, 17]. Two recent DTI studies revealed an abnormal microstructural integrity of the white matter, with higher fractional anisotropy (FA) and lower radial diffusivity (RD) values of the uncinate fasciculus (UF) in the CD male subjects than those in the healthy controls [18, 19]. The UF is a bidirectional, long-range tract of white matter that connects the OFC and the anterior temporal lobes [20]. The abnormal integrity of the UF might get involved in the deficiencies of reversal learning abilities in CD subjects [19], leading to displaying behaviors with negative consequences for both themselves and others, repeatedly [21].

To date, research on CD has focused mainly on male subjects, and few studies have involved female subjects. Thus, little is known about the brain changes of female adolescents with CD. One recent study revealed reduced volumes in the bilateral anterior insula and the right striatal grey matter in female CD patients compared to healthy female controls [22]. When combining male subjects, a significant main effect was found for diagnosis in the right amygdala volume and a significant interaction between sex and diagnosis was found in the anterior insula [22], suggesting that differences of gray matter might exist between males and females with CD. A recent study by Haney-Caron et al. found lower FA and axial diffusivity (AD) values in the anterior/superior corona radiate and the inferior longitudinal and fronto-occipital fasciculi in CD subjects relative to healthy controls, using the tract-based spatial statistics (TBSS) method [23].

Three DTI studies focused on destructive behavior disorder, which includes both CD and oppositional defiant disorder, in adolescents, examining changes in the white matter microstructure in male and female subjects [17, 24, 25]. One of the DTI studies, composed of both males and females, reported that the destructive behavior disorder group had lower FA values in both the frontal and left temporal regions, especially in the left arcuate fasciculus [24], compared to healthy controls. However, the other two studies found no differences between destructive behavior disorder without other comorbidities and healthy control subjects in any DTI index [17, 25]. These divergent results might result from sample heterogeneity, comorbidity with other mental disorders, or different analysis methods [11, 24, 25].

The rate of CD diagnosis in male and female adolescents is about 3 males per 1 female [26]. Despite the lower occurrence of conduct problems in girls, symptoms of confirmed CD in female patients might be more severe than in boys [27]. For example, Hartung et al. reported sex differences in the relation between CD symptoms and disinhibition when the CD subjects performed go/no-go task [28]. Although CD symptoms in boys and girls could not predict disinhibition in the punishment condition, they could predict disinhibition in the mixed-incentive condition in boys [28]. Although several DTI studies have included girls, sex differences in CD were neither examined nor identified clearly in the results. A recent study reported no differences between males and females with CD [23], but the sample size of CD patients was very small (10 males and 7 females) and not homogeneous (both childhood- and adolescent-onset subtypes were included).

In other mental disorders, both Raine et al. and Kim et al. reported reduced volumes of the OFC and the superficial nucleus of the amygdala in men compared to women [29, 30]. Smaller and larger left amygdala volumes in girls and in boys, respectively, were associated with their ability to control emotions [31]. Taken together, these findings suggest that structural differences in the OFC and amygdala might not only be potential risk factors for developing APD in adulthood but could also serve as an explanation for sex differences in APD [29].

Two previous studies have reported abnormalities in the UF in only male CD [18, 19]; therefore, in this study, we recruited well-matched individuals and directly compared microstructural changes of the UF between males with CD and females with CD and between CD females and female healthy controls. Additionally, we aimed to confirm changes in the UF observed between males with CD and healthy males in previous studies [18, 19]. We hypothesized that higher FA and lower RD values would be found in the combined CD group relative to the healthy control group, as no difference in UF between sexes has been reported in the published literature [32].

## 2. Methods

### 2.1. Sample

A total of 27 adolescents with CD (14 males and 13 females, aged from 13 to 16) (see Supplementary Table 1 in Supplementary Material available online at http://dx.doi.org/10.1155/2014/673165) were recruited from outpatient clinics affiliated with the Second Xiangya Hospital of Central South University in Changsha, Hunan, China. To constitute the healthy control group, 29 age-, gender-, and IQ-matched volunteers (16 males and 13 females, aged from 13 to 16) (supplement Table 1) were recruited from a regular school in the same city. The study was approved by the Ethics Committee of the Second Xiangya Hospital of Central South University. All subjects and their parents were aware of the purpose of the study and gave informed written consent.

Diagnoses of CD were made independently by two well-trained psychiatrists, based on the structured clinical interview for the DSM-IV-TR Axis I Disorders-Patient Edition (SCID-I/P) [33]. Psychiatrists rated each symptom item as absent (0), subclinical (1), or clinically present (2), based on the SCID-I/P users' guide, which has been translated into Chinese and adapted for use in both patients and healthy individuals [34]. We did not diagnose CD based solely on information from the adolescent but also interviewed a parent of each subject to obtain detailed information. The psychiatrists made the final judgment if the information offered by the parent and adolescent was not consistent.

For healthy control recruitment, two investigators gave a detailed explanation of the aim and procedure of this research to the headmaster and teachers of the school, in person. Upon obtaining permission from the school administration, students who matched the CD subjects' ages and genders were selected randomly from class rosters. Volunteers who agreed to be interviewed by the psychiatrists were subjected to an SCID-I/P and Wechsler Intelligence Scale for Children-Chinese revision (C-WISC) examinations [35]. Information provided by control subjects was verified by their parents on an as-needed basis. None of the healthy control participants met the criteria for CD.

Exclusion criteria for subjects in both groups were as follows: history of attention deficit hyperactivity disorder, oppositional defiant disorder, any psychiatric or emotional disorder, diagnosis of any pervasive developmental or chronic neurological disorder, Tourette's syndrome, posttraumatic stress disorder, obsessive compulsive disorder, persistent headaches, head trauma, alcohol or substance abuse in the past year, contraindications to magnetic resonance imaging scanning, or an IQ ≤ 80 on the C-WISC. The demographic and clinical characteristics of the subjects are summarized in Table 1. All subjects were right-handed according to the Edinburgh Handedness Inventory [36]. The Chinese version of the Strength and Difficulties Questionnaire (SDQ) [37] was used to detect internalizing and externalizing problems. The Antisocial Process Screening Device (APSD) [38] was used to assess callous-unemotional traits. The presence of callous-unemotional traits is a useful indicator to distinguish different types of CD [39]. All participants with CD were treatment-naïve and fulfilled the criteria for adolescent-onset CD, demonstrating at least one sign of CD after 10 years of age [1].

### 2.2. DTI Acquisition Parameters

Magnetic resonance imaging was performed in a Philips Achieva 3-T scanner with a standard head coil. All participants were asked to remain quiet during scanning. Ear plugs and foam pads were used to minimize noise and head motion. DTI data were acquired using a single-shot spin-echo-planar imaging sequence parallel to the line of the anterior-posterior commissure. The acquisition parameters were as follows: repetition time, 6590 ms; echo time, 70 ms; acquisition matrix, 128 × 128; field of view, 240 mm; slice thickness, 2.5 mm; no gap; and 60 contiguous axial slices. Diffusion-sensitive gradients were applied along 32 noncollinear directions (*b* = 700 s/mm^2^), and one additional image was collected without a diffusion gradient (*b*0 = 0 s/mm^2^). To enhance the signal-to-noise ratio, image acquisition was repeated two times.

### 2.3. DTI Processing

All DTI images were visually screened for abnormal radiological or structural features by a trained technician (ST) during scanning. All acquisitions were checked again using the Statistical Parametric Mapping (SPM8) software (http://www.fil.ion.ucl.ac.uk/spm/) by registering all DTI images to their first *b*0 images. No participants were excluded from further analysis due to excessive head motion (>2.0 mm or 2.0°) [40]. The raw data were analyzed using the brain fMRI software library (FSL, version 5.0, http://www.fmrib.ox.ac.uk/fsl/). Eddy-current correction and head motion correction were performed with fMRIB's Diffusion Toolbox 2.0. FA images were generated with the DTIfit algorithm in the FSL.

After aligning all of the individual FA images to a standard-space template using nonlinear registration, the mean FA image was derived and thinned to generate a mean skeleton that embodied the center of all tracts derived from the whole group. A minimum FA threshold of 0.20 was set to exclude peripheral tracts. Finally, each subject's aligned FA images were projected onto the template skeleton [41]. Individuals' region of interests (ROIs) in the UF were acquired by projecting the Johns Hopkins University white matter atlas onto each subject's native space FA image [42].

**Figure 1:**
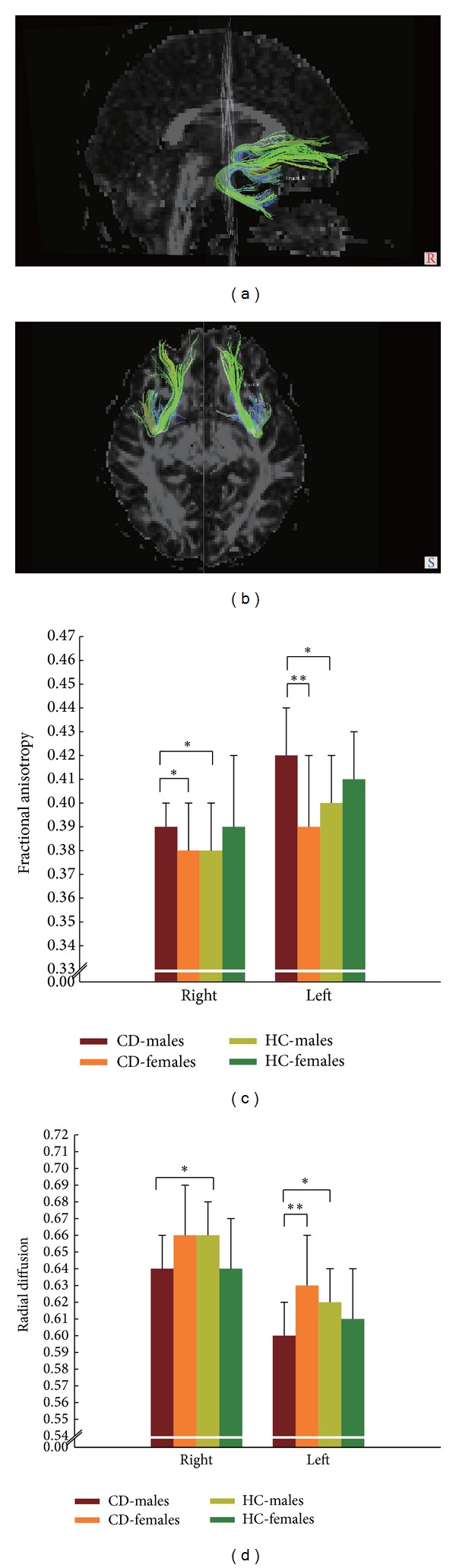
Example of reconstructions of bilateral uncinate fasciculus pathways. (a) Sagittal view and (b) axial view. Comparison of fractional anisotropy (FA) values (c) and radial diffusivity (RD) values (d) in four subgroups. *P < 0.05; **P < 0.01; HC: healthy control.

To confirm the anatomical validity of the UF ROIs used in the analysis, UF tracts were verified in every subject using tractography [42]. Briefly, after head motion and Eddy-current correction, the DTI data were preprocessed with the Diffusion Toolkit (http://www.trackvis.org/dtk/, version 0.6.2.2), and fiber tracking was performed using TrackVis software (http://www.trackvis.org/, version 0.5.2.1). We used the continuous tracking with fiber assignment algorithm (commonly known as FACT) with an angular threshold of 35° to reconstruct the fiber tracts [18]. Fiber tracking of UF was performed using the manual two-ROI approach, a technique shown to identify tracts reliably by the characteristic U-shape of fibers [43] (Figures 1(a) and 1(b)). The integrity of white matter is usually indicated by FA, RD, and MD [44]. FA is the scalar value of anisotropy, representing degree of myelination of fiber bundles. RD measures water diffusive speed perpendicular to the direction of axon, representing the myelin integrity; reduced RD reflects typical maturation of white matter [44]. MD measures the speed of mean diffusion of water but not its directionality. Zahr et al. proposed that the measurements of FA, RD, and MD along the length of a fiber bundle could render estimates of the integrity of white matter [45]. Higher FA and lower RD or MD represents good integrity of white matter [44], which have been found in male CD individuals relative to healthy male controls [18, 19], indicating an atypical maturation of CD individuals' white matter. Additionally, recent research has suggested that other DTI measurements (e.g., AD) might also be able to capture the neurobiologically specific aspects of microstructural abnormality [23]. AD measures water diffusive speed parallel to the direction of axon, reflecting the axonal integrity, and a disproportionate increase of AD might be associated with the axonal damage [44]. Therefore, the FA, MD, AD, and RD values of tracts and the numbers and average lengths of the UF were all calculated to aid the interpretation of any findings in the present study.

### 2.4. Statistical Analysis

All statistical analyses were carried out in SPSS 18.0 (SPSS Inc., Chicago, IL). For the behavioral scale scores, group differences were tested in two-way analysis of variance (ANOVA) with diagnosis and sex as between-group factors. Group differences in the diffusional data (FA, AD, MD, RD, and number and length of fiber bundles) of bilateral UF were analyzed by repeated-measures ANOVAs with hemisphere (left, right) as the within-subjects variable and diagnosis and sex as between-subjects variables. Where significant diagnosis × sex interactions were found, four post hoc contrasts were conducted: (1) CD males versus CD females, (2) CD males versus healthy control males, (3) CD females versus healthy control females, and (4) healthy control males versus healthy control females. A Bonferroni correction was used for multiple comparisons. Then, these data were subjected to Pearson's correlation analysis to assess relationships between behavioral scale scores and DTI measurements, controlling for subject age [19]. *P* < 0.05 was considered to be significant. Mean values are reported with standard deviations.

## 3. Results

The demographic and clinical characteristics of the subjects are summarized in Table 1. ANOVA revealed that the scores for the CD group were higher than those for the healthy control group on the SDQ, conduct problems subscale of the SDQ, the APSD, impulsivity subscale of APSD, and callous-unemotional subscale of APSD (Table 1). For our experiments, the four subgroups were well matched, with no significant differences in terms of age or IQ between the CD male and CD female, CD male and healthy control male, CD female and healthy control female, or healthy control male and healthy control female groups (*P* > 0.05) (supplement Table 1). The scores for conduct problems, impulsivity, and total APSD were significantly higher in the CD male group than in the healthy control male group (*P* < 0.05) (supplement Table 1). Additionally, both the SDQ and APSD total scores and their subscale scores were significantly higher in the CD female group than in the healthy control female group (*P* < 0.05) (supplement Table 1).

For the left and right UF (Figures 1(a) and 1(b)), we did not observe an effect of diagnosis for any diffusional data by repeated-measures ANOVA (*P* > 0.05). However, the FA value was significantly higher, and the MD and RD values were significantly lower [*F* (1, 52) = 41.23, *P* < 0.001 for FA; *F* (1, 52) = 65.69, *P* < 0.001 for MD; *F* (1, 52) = 82.55, *P* < 0.001 for RD, respectively], in the left hemisphere compared to the right hemisphere for all the subjects. Additionally, we observed significant diagnosis × sex interactions in the FA [*F* (1, 52) = 10.35, *P* = 0.002)], MD [*F* (1, 52) = 4.56, *P* = 0.037], and RD [*F* (1, 52) = 9.18, *P* = 0.004].

Post hoc analysis showed that FA values of the bilateral and RD values of the left UF were significantly different between CD male and CD female groups (*P* < 0.05) (Table 2, Figures 1(c) and 1(d)). The FA and RD values of the bilateral UF and the number of fiber bundles of the right UF were significantly different between the CD male and healthy control male groups (*P* < 0.05) (Table 2, Figures 1(c) and 1(d)). Additionally, the RD values of the right UF were different between the CD male and CD female groups at a marginal significance level (*P* = 0.080). The MD values of the right UF were different between the CD male and healthy control male groups at a marginal significance level (*P* = 0.052).

Due to the significant differences between male and female CD subjects on the DTI parameters, the Pearson correlation analysis was performed for male and female subgroups, respectively. For the right UF, significant correlations were found in CD males between RD values and callous-unemotional trait (*r* = −0.60, *P* = 0.030) (uncorrected, *P* < 0.05), between MD values and callous-unemotional trait (*r* = −0.60, *P* = 0.031) (uncorrected, *P* < 0.05), and between the number of fiber bundles and APSD (*r* = 0.58, *P* = 0.037) (uncorrected, *P* < 0.05). For the left UF, correlations between FA values and callous-unemotional trait (*r* = −0.52, *P* = 0.071), between FA values and APSD (*r* = −0.54, *P* = 0.055), and between MD values and SDQ (*r* = −0.53, *P* = 0.074) were only marginally significant (uncorrected, *P* < 0.05) in CD males. No significant correlations were found between DTI indices and behavioral scores in the female CD group.

## 4. Discussion

To the best of our knowledge, this is the first report of the differences in white matter microstructural integrity between CD males and CD females by using tractography. Significantly higher FA and lower RD values were found in the CD male group compared to the CD female group, contrasting to Haney-Caron et al. negative finding between male and female CD individuals by TBSS [23]. Our results also replicated the findings by Passamonti et al and Sarkar et al., who reported higher FA and lower RD values in CD males compared to healthy control males [18, 19].

The FA measures the degree of the diffusion in different directions, which depends on the size, density, and organization of the axons, as well as the degree of myelination and number of neural branches per imaging voxel. The RD is selectively sensitive to the membrane permeability and myelin damage. Higher FA and lower RD or MD are commonly used indicators of good integrity of fiber bundles [44]. Thus, the observation of increased FA with decreased RD or MD suggests that there might be healthier axonal integrity and greater myelination in males with CD compared to the females with CD, as well as the healthy male controls.

Previous DTI studies on CD boys attributed higher FA and lower RD values in the UF to deficiencies of reversal learning abilities, which refers to learning to “reverse” responses that were previously rewarded but later punished [18–20]. Difficulties in reversal learning contribute to the perseveration of antisocial behavior in both boys with CD or psychopathy, as these boys could not learn to avoid behaviors that have negative consequences for themselves and others [18, 19, 21, 29]. Herpertz et al. reported a lower level of response to aversive emotional stimuli in CD subjects, which might result from deficits in an associative information processing system, which produces adaptive cognitive-emotional reactions in normal individuals [46]. Additionally, the higher FA and lower RD values in CD males compared to CD females shown in our results might be due to the reduced ability of reversal learning in CD males. However, further studies on larger samples will reveal the potential reasons for gender differences in CD. Nonetheless, our result demonstrating higher FA and lower RD values in CD males compared to CD females provides a primary clue to why boys have higher rates of conduct problems than girls.

A low social emotional processing ability has been reported in CD subjects [26], which has been used to account for gender differences in the disinhibition of the mixed-incentive condition in a go/no-go task [28]. The UF is associated with the function of social emotional processing in humans [20]. Thus, the higher FA and lower RD values in the male CD group compared to the female CD group in our results might suggest the males have poor social emotional processing ability. Similar results have been reported in previous DTI studies on CD boys [18, 19]. The UF is also associated with language function [20]. Verbal deficits have been regarded as one of the most relevant factors in the different susceptibility for male and female subjects to develop CD [47]. Accordingly, gender differences in our results might also be associated with less vulnerability to verbal deficits in female individuals.

Caspi et al. demonstrated that low activity in the promoter of the monoamine oxidase A (MAOA) gene might be one risk factor for boys who are susceptible to CD [48]. The MAOA gene is located in the X chromosome and displays either high activity (MAOA-H) or low activity (MAOA-L) in male subjects. Female subjects have two X chromosomes and, thus, can be present in at least three subgenotypes (HH, HL, and LL), resulting in a lower occurrence of CD in females [49]. Our results showing lower FA and higher RD values in female CD subjects might be due to the effects of MAOA—a hypothesis that will need to be studied further in future genetic research.

The UF connects the PFC and the temporal lobe; therefore, its information transmission properties can be predicted by the function of its connecting regions [20]. Impairment of the PFC and the amygdala in CD individuals has been reported (see review by Sterzer and Stadler, 2009). The PFC and amygdala have been implicated in emotional processing and behavior regulation [50]. For example, in sMRI studies, Ameis et al. reported a thinner cortex thickness of the left OFC. Additionally, the thickness of the right medial temporal cortex was negatively correlated with external behaviors in healthy subjects [51]. Bobes et al. reported functional and structural abnormalities in the left amygdala of reactive aggression men [52].

In fMRI studies, Viding et al. reported a greater response to fearful faces in the right amygdala in subjects who had CD without the callous-unemotional trait compared to subjects who had CD with the callous-unemotional trait [15]. In a task of repeatedly viewing violent media materials by healthy individuals, Kelly et al. reported lower response in the right OFC and a lower interaction between the OFC and the amygdala [53]. Both Rubia et al. and Finger et al. reported reduced activation of the amygdala in the interference inhibition task and reduced reactivation of the OFC during the reward task in CD compared to healthy controls [10, 11, 54]. The results presented in these studies could be due to abnormalities in the orbitofrontal-paralimbic motivation networks in CD subjects, which serve as a potential factor for the repeated impulsive and disadvantageous decisions shown by CD subjects.

As suggested by Hyde et al. in their latest review on antisocial behavior disorder, the interpretation of results about CD should consider the age of CD onset and the comorbidity of the callous-unemotional trait [26]. Our results demonstrated that FA values were not only higher in the left UF, similar to CD with callous-unemotional trait [19], but also higher in the right UF in CD without callous-unemotional trait males. In combination with the report on the childhood-onset subtype of CD by Passamonti et al. [18], our results indicate that the phenomenon of higher FA and lower RD also exists in adolescence-onset subtype of CD males.

However, our results were somewhat different from previous reports. Using TBSS, Haney-Caron et al. found no significant gender difference in any fiber bundles in CD [23]. Their sample included both childhood- and adolescent-onset subtype CD. Although no significant differences were found between the two subtypes in their study, significant differences of brain structures had previously been reported in a larger sample [6, 55]. Different analysis methods might also account for the divergence between the findings of our report and those of Haney-Caron et al. [23]. TBSS mainly skeletonizes the FA map and presents the highest portions of the skeleton, whereas tractography analysis encompasses the white matter tract following specific directions [56]. Additionally, our samples ranged in age from 13 to 16 years, whereas Sarkar et al.'s ranged from 12 to 19 years, and Passamonti's were about 18.5 years old [18, 19]. There is a rapid maturation period for the structure and function of gray matter and white matter during adolescence [26], which might account for our results showing bilaterally higher UF FA values in CD males rather than in one hemisphere, as shown in previous studies [18, 19].

There are several potential limitations in this study. First, subjects with high callous-unemotional traits should be included in future studies, as the presence of callous-unemotional traits has been used to distinguish different types of CDs. Second, the sample sizes were moderate, which might affect the statistical power of this study. Additionally, the multiple correlations were only exploratory analysis due to the limited sample sizes. Therefore, bigger sample sizes will be needed in the future to confirm the present results with more statistically stringent significant level.

In summary, our results replicated previous findings demonstrating that there is abnormal maturation of the UF in CD males. Additionally, we showed significant effects of gender on the integrity of the bilateral UF in CD individuals. This result may indicate why boys have higher rates of conduct problems than girls.

## Supplementary Material


The four subgroups were well matched, with no significant differences in terms of age or IQ between the CD male and CD female, CD male and healthy control male, CD female and healthy control female, or healthy control male and healthy control female groups (*p* > 0.05). The scores for conduct problems, impulsivity, and total APSD were significantly higher in the CD male group than in the healthy control male group (*p* < 0.05). Additionally, both the SDQ and APSD total scores and their subscale scores were significantly higher in the CD female group than in the healthy control female group (*p* < 0.05).

## Figures and Tables

**Table 1:** Demographic and clinical characteristics of the participants (mean ± S.D).

	Conduct disorder (*n* = 27)	Healthy control (*n* = 29)	*F* value	*P* value
Age	14.1 ± 0.8	14.4 ± 0.7	2.36	0.130
IQ	104 ± 11	106 ± 6	0.78	0.381
Conduct problems (SDQ)	4.7 ± 1.7	2.2 ± 1.2	16.66	<0.001**
Total problems (SDQ)	16.7 ± 5.7	10.9 ± 5.1	13.92	<0.001**
Impulsivity (APSD)	5.0 ± 2.0	3.0 ± 1.5	14.40	<0.001**
Callous-unemotional traits (APSD)	5.8 ± 2.2	3.8 ± 1.5	5.42	0.024*
Total score (APSD)	16.0 ± 3.6	9.5 ± 2.0	34.20	<0.001**

SDQ: Strength and Difficulties Questionnaire; APSD: the Antisocial Process Screening Device; *P = 0.05; **P = 0.01.

**Table 2:** DTI measurement of the four subgroups (Mean ± S.D.).

	Conduct disorder		Healthy control group	
	Male (*n* = 14)	Female (*n* = 13)	Male (*n* = 16)	Female (*n* = 13)
FA (right)	0.39 ± 0.01^a,b^	0.38 ± 0.02	0.38 ± 0.02	0.39 ± 0.03
MD (right)	0.82 ± 0.02	0.84 ± 0.03	0.84 ± 0.02	0.83 ± 0.02
AD_(right)	1.20 ± 0.03	1.20 ± 0.04	1.21 ± 0.03	1.21 ± 0.04
RD_(right)	0.64 ± 0.02^b^	0.66 ± 0.03	0.66 ± 0.02	0.64 ± 0.03
Length_(right)	75 ± 11	70 ± 15	75 ± 14	73 ± 12
Number_(right)	315 ± 73^b^	254 ± 117	232 ± 130	258 ± 125

FA (left)	0.42 ± 0.02^a,b^	0.39 ± 0.03	0.40 ± 0.02	0.41 ± 0.02
MD (left)	0.80 ± 0.02	0.82 ± 0.03	0.81 ± 0.02	0.81 ± 0.02
AD (left)	1.20 ± 0.03	1.18 ± 0.03	1.20 ± 0.04	1.20 ± 0.03
RD (left)	0.60 ± 0.02^a,b^	0.63 ± 0.03	0.62 ± 0.02	0.61 ± 0.03
Length (left)	71 ± 8	70 ± 12	74 ± 13	66 ± 15
Number (left)	194 ± 82	249 ± 134	176 ± 99	200 ± 108

Note: ^a^group means differ significantly from the female CD group at *P* < 0.05 after Bonferroni correction; ^b^group means differ significantly from the male healthy control group at *P* < 0.05 after Bonferroni correction; FA: fractional anisotropy; MD: mean diffusivity; AD: axial diffusivity; RD: radial diffusivity. MD, AD, and RD values and S.D. ×10^−3^ mm^2^/s.
