# Supplementary material for: Sex Differences of Uncinate Fasciculus Structural Connectivity in Individuals with Conduct Disorder
Source: Biomed Res Int. 2014 Apr 14;2014:673165. doi: 10.1155/2014/673165 (PMC4009134; doi:10.1155/2014/673165)
Supplement: Supplementary file 1 — The four subgroups were well matched, with no significant differences in terms of age or IQ between the CD male and CD female, CD male and healthy control male, CD female and healthy control female, or healthy control male and healthy control female groups (p > 0.05). The scores for conduct problems, impulsivity, and total APSD were significantly higher in the CD male group than in the healthy control male group (p < 0.05). Additionally, both the SDQ and APSD total scores and their subscale scores were significantly higher in the CD female group than in the healthy control female group (p < 0.05). [file 673165.f1.pdf]

Supplement

Table 1. Demographic and clinical characteristics of the four subgroups (Mean ± S.D.).

	**Conduct Disorder**		**Healthy Control**	
	**Male (n = 14)**	**Female (n = 13)**	**Male (n = 16)**	**Female (n = 13)**
Age	14.3 ± 0.7	13.9 ± 0.8	14.6 ± 0.7	14.2 ± 0.6
IQ	103 ± 12	105 ± 10	107 ± 6	105 ± 7
Conduct problems (SDQ)	4.5 ± 2.1[a]	4.5 ± 1.6[b]	2.6 ± 1.3	1.8 ± 0.8[c]
Total problems (SDQ)	14.9 ± 6.2	18.9 ± 4.2[b]	12.3 ± 5.9	9.4 ± 3.4
Impulsivity(APSD)	4.6 ± 2.0[a]	5.4 ± 1.9[b]	2.9 ± 1.6	3.1 ± 1.5
callous-unemotional traits (APSD)	5.8 ± 2.7	5.8 ± 1.5[b]	4.3 ± 1.0	3.3 ± 1.8
Total score(APSD)	15.1 ± 3.7[a]	17.1 ± 3.4[b]	10.0 ± 1.6	8.9 ± 2.3

Note：[a] group means differ significantly from the male healthy control group at $p < 0.05$ after Bonferroni correction; [b] group means differ significantly from the female healthy control group at $p < 0.05$ after Bonferroni correction; [c] group means differ significantly from the male healthy control group at $p < 0.05$ after Bonferroni correction. SDQ = the Strength and Difficulties Questionnaire; APSD = the Antisocial Process Screening Device.
